# Riboflavin intake and kidney health: population evidence and mechanistic insights from NHANES and molecular docking analyses

**DOI:** 10.1080/0886022X.2025.2611520

**Published:** 2026-01-25

**Authors:** Peiwen Wu, Wenjie Long, Xiang Xiao, Li Wang, Ying Xu, Xin Ma

**Affiliations:** a Department of Clinical Medicine, School of Clinical Medicine, Chengdu Medical College, Chengdu, China; b Department of Nephrology, The First Affiliated Hospital of Chengdu Medical College, Chengdu, China; c Health Management Centre, The First Affiliated Hospital of Chengdu Medical College, Chengdu, China; d Outpatient Department, The First Affiliated Hospital of Chengdu Medical College, Chengdu, China; e Sichuan Clinical Research Center for Geriatrics, The First Affiliated Hospital of Chengdu Medical College, Chengdu, China

**Keywords:** Riboflavin, chronic kidney disease, NHANES, nutrition epidemiology, oxidative stress, mitochondrial metabolism

## Abstract

Chronic kidney disease (CKD) constitutes a significant global public health challenge, with emerging evidence suggesting that riboflavin may confer renoprotective effects. However, the association between dietary riboflavin intake and CKD risk remains inadequately elucidated. To investigate this, we analyzed National Health and Nutrition Examination Survey (NHANES) 2005–2018 datasets using weighted multivariate logistic regression, interaction testing, stratified subgroup analyses, and curve fitting. Additionally, network pharmacology and molecular docking simulations were employed to identify and validate potential therapeutic targets. Our results showed that patients with CKD exhibited significantly lower riboflavin intake than their non-CKD counterparts (1.98 vs. 2.21 mg/d, *p* < 0.001). After full adjustment, each 1 mg/d increment in riboflavin intake was associated with an 18.8% reduced risk of CKD (adjusted OR = 0.812, 95% CI: 0.686–0.962). Individuals in the highest intake quartile had a 42.7% lower risk compared to the lowest quartile (adjusted OR = 0.573, 95% CI: 0.400–0.822). A non-linear dose-response relationship was observed, characterized by an inflection point at 1.66 mg/d, indicating a more pronounced protective effect at lower intake levels. Mechanistic investigations suggested that riboflavin’s benefits may be mediated through interactions with key targets like caspase-3 (CASP3), Erb-B2 receptor tyrosine kinase 2 (ERBB2), and matrix metallopeptidase 9 (MMP9), implicating apoptotic and metabolic pathways. In conclusion, dietary riboflavin intake is inversely associated with CKD risk, particularly at lower concentrations, and strategic augmentation of intake represents a promising dietary intervention for CKD prevention and management.

## GRAPHICAL ABSTRACT

**Figure:**
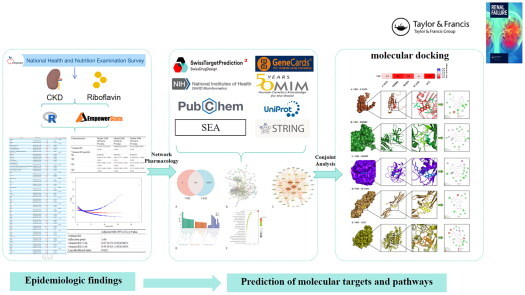

## 1. Introduction

Chronic kidney disease (CKD) constitutes a notable global public health challenge, marked by elevated incidence and mortality rates [1]. This pathophysiological entity is demarcated by compromised renal functional capacity and a relentless deterioration in glomerular filtration rate (GFR) [2], precipitating not only the disintegration of normative nephric physiology but also propagating deleterious multisystem repercussions across cardiovascular, osseous, and immunological domains [3,4]. Notwithstanding considerable heterogeneity in global prevalence estimates for CKD, its substantive disease burden is universally acknowledged. Contemporary epidemiological assessments indicate that approximately one-tenth of the adult population worldwide is afflicted by CKD, encompassing an aggregate exceeding 500 million individuals [5]. Another study shows that there are 674 million CKD patients worldwide, equivalent to an average of one in 10 adults [6]. Concomitantly, current projections derived from the World Population Prospects dataset indicate that by mid-century, virtually every major global region will surpass the threshold wherein at least one-fourth of its inhabitants attain or exceed 60 years of age [7]. Given the exponential trajectory of the prevalence of both CKD and end-stage kidney disease (ESKD) with increasing age, CKD emerges as a profound and increasingly urgent public health issue of global significance, affecting hundreds of millions globally, particularly in aging populations.

The pathogenetic trajectory of CKD is orchestrated by a constellation of multifactorial determinants, encompassing demographic stratifications (age, sex, and ethnicity), socioeconomic gradients, and behavioral paradigms typified by lifestyle aberrations and poor nutrition modalities. Within male cohorts, CKD progression is principally propelled by hypertensive and diabetic etiologies, with disease incidence demonstrating marked ascension beyond the quinquagenarian threshold [8]. Conversely, the female vulnerability profile is principally governed by autoimmune pathophysiologies (exemplified by lupus nephritis) and recurrent urinary tract infections [9], further potentiated through accelerated renal fibrogenesis consequent to estrogen diminution in the postmenopausal phase [10]. Ethnic disparities manifest distinctly in both the prevalence and disease trajectories of CKD, as underscored by a Singaporean cohort study delineating divergent susceptibility patterns among Chinese, Malay, and Indian populations [11]. Furthermore, individuals occupying disadvantaged socioeconomic strata manifest amplified health susceptibilities, consequently potentiating CKD risk [9]. Elucidating these multifactorial risk stratifications proves imperative for devising precision-targeted preventive frameworks and interventional modalities directed at mitigating CKD incidence and decelerating pathological progression.

The contemporary therapeutic paradigm for CKD is predicated upon a tripartite prevention and control framework comprising primary prevention initiatives, early diagnostic identification coupled with therapeutic intervention, and comprehensive management of disease sequelae [12]. Notwithstanding, formidable clinical impediments endure, encompassing adverse pharmacotherapeutic sequelae (e.g., RAAS inhibitor-induced hyperkalemia), inequitable dialysis resource allocation (accessible to a mere decile of the global patient cohort), and a critical organ transplantation deficit (exhibiting a 1:10 supply–demand disproportion). Conversely, dietary and nutritional interventions manifest as a superior risk-mitigation paradigm for CKD, owing to their salubrious safety profile, augmented accessibility, and intrinsic capacity for polypharmacological modulation.

Vitamin B2, conventionally designated as riboflavin, exhibits ubiquitous distribution across a spectrum of dietary reservoirs encompassing dairy products, ova, piscine sources, animal proteins, oleaginous seeds, and cruciferous cultivars. This indispensable micronutrient serves as a cardinal renoprotective agent, principally mediated through a tripartite mechanistic framework: antioxidative defense systems, metabolic homeostasis modulation, and antifibrotic bioactivity. Specifically, riboflavin operates in its coenzymatic incarnations FAD (flavin adenine dinucleotide) and FMN (flavin mononucleotide) to orchestrate redox cascades, perpetuate the glutathione redox cycle, and neutralize reactive oxygen species (ROS), consequently attenuating oxidative stress-induced tissue damage [13]. Experimental animal models have evidenced that riboflavin deficiency induces mitochondrial dysfunction and potentiates glomerulosclerotic progression within the pathophysiological milieu of diabetic nephropathy [14]. Riboflavin participates in fatty acid β-oxidation, improves renal energy metabolism disorders, and inhibits lipid toxicity-induced podocyte apoptosis [15]. Mechanistically, riboflavin mitigates renal interstitial fibrogenesis through modulation of the TGF-β/Smad3 signaling axis, thereby attenuating collagen deposition [16–18]. However, despite its crucial role in cellular metabolism, riboflavin remains a relatively underexplored nutrient in epidemiological studies of CKD risk and progression. Future studies need to further investigate the specific effects of dietary riboflavin on kidney function in CKD patients, as well as the optimal riboflavin supplementation dose.

Building upon this evidentiary foundation, the present investigation pioneers the integration of population-level epidemiological data from the National Health and Nutrition Examination Survey (NHANES) with multi-tiered omics methodologies, thereby pursuing three cardinal objectives: to delineate the epidemiological nexus between dietary riboflavin intake and CKD susceptibility among US adults; to identify candidate therapeutic targets of riboflavin through systematic network pharmacology interrogation, with parallel validation of ligand-target binding affinities at core nodes via molecular docking simulations; and to provide evidence-based evidence and theoretical basis for the prevention and treatment of CKD through nutrition.

## 2. Materials and methods

### 2.1. Study design and data source

NHANES represents a nationally representative stratified multistage probability sample survey administered by the National Center for Health Statistics (NCHS) [19]. The data from participants in the NHANES database primarily includes demographic information, dietary information, examination data, laboratory data, and other information such as lifestyle and health status. The NHANES survey uses a stratified multistage probability sampling design to ensure the representativeness of the non-institutionalized civilian population in the United States. The data utilized in this study comprise information derived from structured household interviews and physical assessments administered at Mobile Examination Centers (MECs). Approval for the study protocol was granted by the NCHS Research Ethics Review Committee, with written informed consent secured from all participants prior to their enrollment. This study adheres to the STROBE reporting guidelines to enhance the reporting quality of epidemiological observational studies. NHANES constitutes a publicly accessible database where participant information is de-identified; consequently, informed consent and ethical approval are not requisite for this study. For additional details regarding the NHANES database, refer to http://www.cdc.gov/nhanes.

### 2.2. Study population

**Figure 1.**
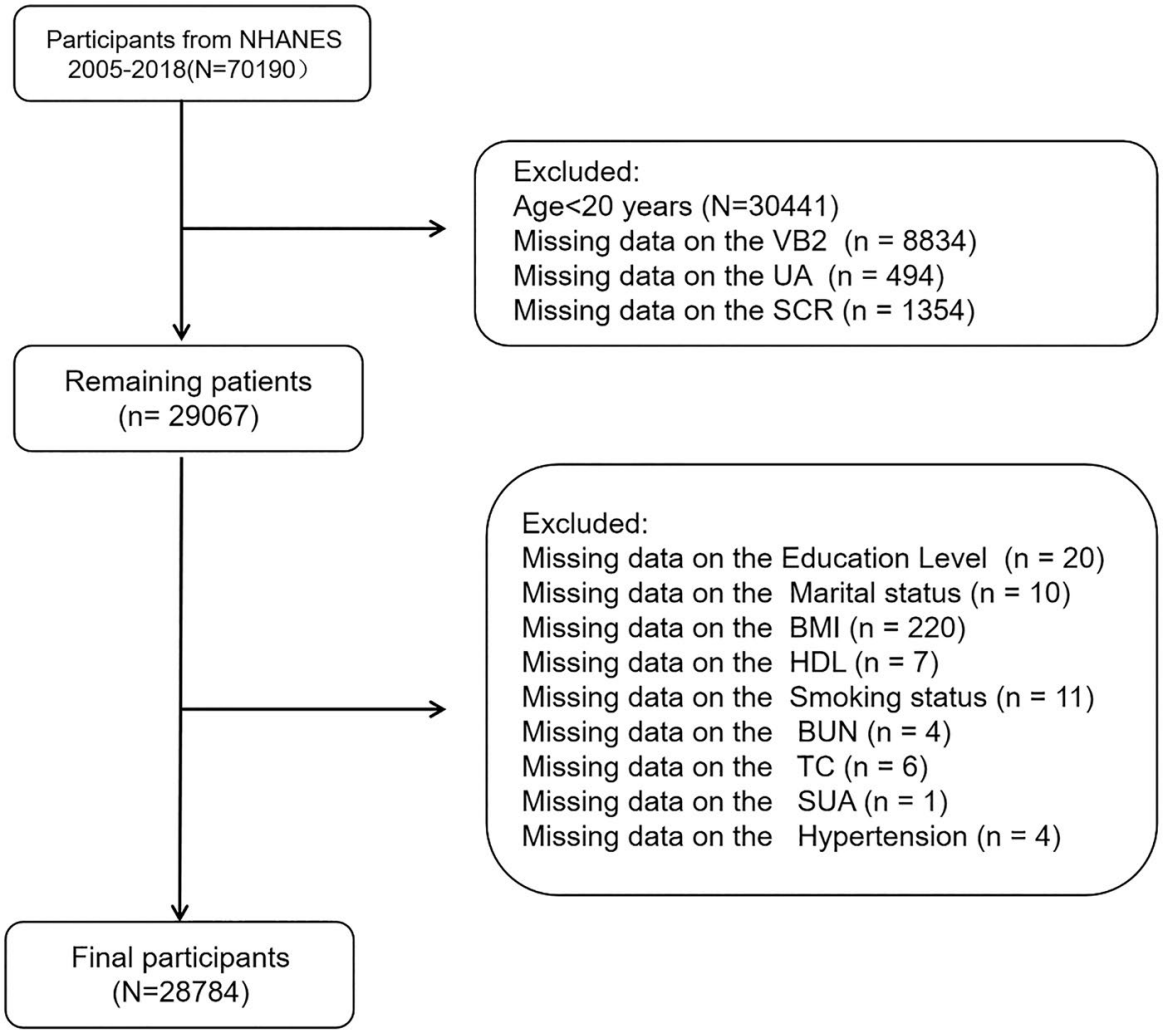
Flow diagram of participant selection. Vitamin B2 (VB2); urinary levels of albumin (UA); serum creatinine (SCR); body mass index (BMI); high-density lipoprotein cholesterol (HDL); urea nitrogen (BUN); total cholesterol (TC) and uric acid (SUA).

This cross-sectional investigation leveraged population-based data from the NHANES spanning 2005–2018, initially comprising 70,190 participants. Implementation of rigorous inclusion/exclusion criteria yielded a final analytical cohort of 28,784 U.S. adults derived from NHANES cycles 2005–2018. Figure 1 delineates the participant selection workflow, executed through comprehensive application of established screening and exclusion protocols as documented in contemporary epidemiological literature.

### 2.3. Study variable

#### 2.3.1. Result variable

CKD is defined per the Kidney Disease: Improving Global Outcomes (KDIGO) Clinical Practice Guideline for Glomerular Diseases. Diagnostic confirmation necessitates meeting at least one criterion persisting for ≥3 months: either (1) a sustained reduction in estimated glomerular filtration rate (eGFR) below 60 mL/min/1.73 m^2^, calculated via the CKD Epidemiology Collaboration (CKD-EPI) creatinine equation or (2) urinary albumin-to-creatinine ratio (UACR) levels surpassing the diagnostic threshold of 30 mg/g [20].

#### 2.3.2. Exposed variable

Data regarding vitamin B2 consumption were ascertained through 24-h dietary recall interviews administered across two discrete temporal intervals: the primary assessment was conducted via in-person methodology at the MEC, whereas the secondary evaluation was implemented telephonically 3–10 days subsequently. The dietary interview section of the NHANES website provides a comprehensive overview of the interview procedure [21]. Dietary intake levels of vitamin B2 were calculated as the mean value derived from the aggregate nutrient intakes documented during these two 24-h assessments.

#### 2.3.3. Covariates

This investigation incorporated several covariates to mitigate potential confounding effects, selected based on their established associations with both the exposure and outcome variables. These included: age; sex; race; education level; marital status; body mass index (BMI); the poverty income ratio (PIR); serum concentrations of triglycerides (TGs), total cholesterol (TC), high-density lipoprotein cholesterol (HDL-C), low-density lipoprotein cholesterol (LDL-C), serum uric acid (SUA), blood urea nitrogen (BUN), and serum creatinine (SCR); urinary levels of albumin (UA) and urinary creatinine (UCr); vitamin B6 (VB6), vitamin B12 (VB12), magnesium (Mg), calcium (Ca), total energy, and total fat; smoking status (defined dichotomously as: YES: lifetime consumption exceeding 100 cigarettes; NO: lifetime consumption of ≤100 cigarettes); diabetes (defined by the presence of at least one criterion: (1) documented medical history of diabetes, (2) current use of glucose-lowering agents, or (3) hemoglobin A1c (HbA1c) level ≥6.5%; and hypertension (ascertained according to the 2017 American College of Cardiology/American Heart Association clinical practice guidelines, defined as: average diastolic blood pressure ≥80 mmHg and/or average systolic blood pressure ≥130 mmHg, a documented history of hypertension, or current use of antihypertensive medication).

### 2.4. Network pharmacological analysis

The mechanistic foundations underpinning riboflavin’s nephroprotective properties were elucidated through integrated network pharmacology interrogation. Primarily, the compound’s two-dimensional/three-dimensional structural configurations and SMILES notation were acquired from the PubChem repository (https://pubchem.ncbi.nlm.nih.gov). This molecular descriptor was subsequently inputted into dual predictive architectures – the SwissTargetPrediction server (http://www.swisstargetprediction.ch/) [22] and the Similarity Ensemble Approach (SEA) server (https://sea.bkslab.org/) [23] – to prognosticate putative molecular targets. To optimize predictive fidelity, under the ‘Homo sapiens’ condition the targets were screened according to probability ≥0 and Max Tc ≥0.25. Researchers further corrected the predicted targets through the Uniprot (https://www.uniprot.org/) database to obtain gene information on drug targets. Concurrently, pathologically relevant targets associated with CKD were systematically curated from the GeneCards (https://www.genecards.org/) and OMIM (https://omim.org/) repositories. Subsequently, the predicted targets of riboflavin were matched and mapped with protein targets associated with CKD. The common targets identified were determined to be potential targets for riboflavin treatment of CKD. Next, protein–protein interactions (PPIs) of overlapping genes were analyzed in the STRING database (http://string-db.org/), and a PPI network was constructed using Cytoscape (Version 3.8.2) software. Additionally, hub genes were screened using the MCODE plugin (Version 1.6.1) in Cytoscape (Version 3.7.1). Finally, the identified core targets were imported into the Kyoto Encyclopedia of Genes and Genomes (KEGG) and Gene Ontology (GO) analysis, annotation, visualization, and integrated discovery database (DAVID, http://david.abcc.ncifcrf.gov/). GO terms and KEGG pathways with *p* values less than 0.05 were considered significant.

### 2.5. Molecular docking

The two-dimensional structural representation of riboflavin was acquired from the PubChem and ZINC chemical repositories, and used Avogadro software to model the ligand and optimize its framework energy. The optimized structure was saved in PDB format. Then, AutoDockTools-1.5.6 was used to add charges and identify rotatable bonds, and the file was finally saved in PDBQT format.

Leveraging the PDB repository (https://www.rcsb.org/), we procured crystalline structures of proteins encoded by hub genes. These macromolecular configurations were subsequently subjected to structural refinement in Chimera 1.17.3, involving the excision of non-essential ligands and solvent molecules. Following preparatory optimization, polar hydrogen atoms were computationally integrated utilizing AutoDockTools-1.5.6, with resultant topologies archived in PDBQT format. Then, explore the active pockets of these proteins by adjusting the *X*–*Y*–*Z* coordinates and grid size of these proteins for molecular docking studies. In addition, the relevant binding sites were optimized. AutoDock Vina was used to perform 10 docking analyses on the active compounds and proteins of interest, and the minimum binding energy of each round of analysis was recorded as the final value.

### 2.6. Molecular dynamics simulation

Employing GROMACS 2020 [24], a 100-nanosecond molecular dynamics (MD) simulation was executed to elucidate the conformational dynamics of the ligand–protein complex. The target protein’s crystallographic structure (PDB file) underwent rigorous structural interrogation utilizing Chimera 1.17.3 [25]. Protein files were created using the Amber99SB force field, which helps to more accurately describe the interactions between amino acids. Similarly, ligand files were generated using softtop 1.0 software to ensure the correct representation of ligands in the system. The simulation system was constructed as a cubic box solvated with SPC water molecules under periodic boundary conditions. System charge was neutralized by adding sodium chloride (NaCl) ions. Energy minimization was performed for 10,000 steps using the steepest descent algorithm followed by the conjugate gradient method. The system was then equilibrated to the target temperature and pressure to mimic physiological conditions. Subsequently, a 100 ns production run was conducted under standard conditions, with a time step of 2 fs (totaling 1,000,000 steps). Trajectory data were recorded every 30 ps. Finally, the binding free energy between the receptor and ligand was calculated using the MM/PBSA approach.

### 2.7. Statistical considerations

All analyses accounted for the complex, multi-stage probability sampling design of NHANES to ensure national representativeness and accurate variance estimation. This was implemented by incorporating the strata (SDMVSTRA), primary sampling units (SDMVPSU), and the two-year MEC weights (WTMEC2YR) for the combined 2005–2018 survey cycles, in accordance with NCHS analytical guidelines. This complex survey design was explicitly specified in all weighted multivariate logistic regression models and subgroup analyses.

Missing data for the primary exposure (dietary riboflavin), outcome (CKD status), and essential covariates were handled via listwise deletion, with the participant flow detailed in Figure 1.

We employed weighted multivariable logistic regression to evaluate the association between riboflavin intake and CKD. The selection of covariates was determined *a priori* based on a comprehensive review of the literature, aiming to account for known or suspected common causes of both riboflavin intake and CKD risk, thereby minimizing confounding bias. We deliberately chose not to include composite dietary pattern indices (e.g., HEI, DASH) as primary covariates in our main analysis to avoid potential over-adjustment bias. Since riboflavin is an intrinsic component of overall diet quality, adjusting for a composite index might inadvertently adjust away part of the exposure’s effect. To assess the potential for confounding by overall dietary structure, we performed a sensitivity analysis stratifying by total protein intake, a key component that serves as a practical proxy for broader dietary patterns.

### 2.8. Statistical analyses

Comprehensive descriptive analytics were executed across all study participants utilizing Empower Stats (v4.2) (Helsinki, Finland) and R software (v4.4.2) (R Foundation for Statistical Computing, Vienna, Austria), employing a statistical significance threshold of *p* < 0.05. Investigational data underwent calibrated survey weighting to maintain national representativeness. Participant stratification delineated baseline characteristic cohorts according to CKD status. Continuous variables were articulated as mean ± standard error, while categorical variables are expressed as percentages. Weighted multivariate logistic regression modeling was deployed to interrogate the association between CKD status and dietary vitamin B2 intake, with outcomes quantified as odds ratios (ORs) accompanied by 95% confidence intervals (95% CIs). Three hierarchical multivariable models were formulated: model 1 no adjusted; model 2 incorporating demographic covariate adjustments (encompassing age, sex, marital status, education level, PIR, and race); and model 3 further integrating comprehensive clinical covariate adjustments (including age, sex, marital status, education level, PIR, race, BMI, smoking status, TG, TC, HDL-C, LDL-C, SUA, SCR, BUN, UA, UCr, hypertension, diabetes, VB6, VB12, Ca, total energy, and total fat). Stratified subgroup analyses were executed across diverse demographic and clinical stratifying variables. Formal tests for interaction were performed using likelihood-ratio tests by introducing multiplicative interaction terms (dietary riboflavin intake × subgroup variable) into the multivariable logistic regression models. Multivariable regression models featuring covariate-adjusted flexible smoothing curves were constructed to delineate potential nonlinear relationships. Subsequent threshold effect analyses were conducted to elucidate potential transition points within the CKD–riboflavin association continuum.

## 3. Results

### 3.1. Baseline characteristics

A total of 28,784 participants met the study criteria and were included in the final analysis. Table 1 delineates the baseline characteristics of the cohort stratified by CKD status, with 5,425 individuals assigned to the CKD group. The overall cohort exhibited a mean age of 61.11 years, comprising 2,871 male participants. Comparative analysis revealed statistically significant differences (*p* < 0.05) between the CKD and non-CKD groups across multiple parameters: age; race; education level; marital status; PIR; BMI; EGFR; UACR; smoking status; hypertension and diabetes; urinary biomarkers (specifically on-site collected UA and UCr); serum biomarkers including TG, LDL-C, SUA, BUN, and SCR; as well as dietary intake profiles encompassing Ca, Mg, VB6, VB12, total energy, and total fat.

**Table 1.** Baseline characteristics of the study participants from NHANES 2005–2018, stratified by chronic kidney disease (CKD).

	No CKD (*n* = 23,359)	With CKD (*n* = 5,425)	*p* Value
Age	45.59 (45.15, 46.04)	61.11 (60.40, 61.83)	<0.0001
Sex			0.322
Male	11,171 (47.8%)	2,554 (47.1%)	
Female	12,188 (52.2%)	2,871 (52.9%)	
Race			<0.001
Mexican American	3,814 (16.3%)	642 (11.8%)	
Other Hispanic	2,319 (9.9%)	386 (7.1%)	
Non-Hispanic White	10,198 (43.7%)	2,587 (47.7%)	
Non-Hispanic Black	4,531 (19.4%)	1,444 (26.6%)	
Other race – including multi-racial	2,497 (10.7%)	366 (6.7%)	
Education level			<0.001
Less than 9th grade	2,025 (8.7%)	703 (13.0%)	
9–11th grade (including 12th grade with no diploma)	3,039 (13.0%)	875 (16.1%)	
High school graduate/GED or equivalent	5,252 (22.5%)	1,344 (24.8%)	
Some college or AA degree	7,122 (30.5%)	1,513 (27.9%)	
College graduate or above	5,921 (25.3%)	990 (18.2%)	
Marital status			<0.001
Married	12,508 (53.5%)	2,752 (50.7%)	
Widowed	1,182 (5.1%)	990 (18.2%)	
Divorced	2,425 (10.4%)	696 (12.8%)	
Separated	754 (3.2%)	196 (3.6%)	
Never married	4,432 (19.0%)	537 (9.9%)	
Living with partner	2,058 (8.8%)	254 (4.7%)	
PIR	3.06 (3.00, 3.13)	2.73 (2.64, 2.81)	<0.0001
BMI			<0.001
≤25	6,838 (29.27%)	1,254 (23.12%)	
>25, ≤30	7,832 (33.53%)	1,717 (31.65%)	
>30	8,689 (37.20%)	2,454 (45.24%)	
Smoking status			<0.001
No	13,249 (56.7%)	2,733 (50.4%)	
Yes	10,110 (43.3%)	2,692 (49.6%)	
Hypertension			<0.001
No	12,346 (52.9%)	1,128 (20.8%)	
Yes	11,013 (47.1%)	4,297 (79.2%)	
Diabetes			<0.001
No	20,685 (88.6%)	3,492 (64.4%)	
Yes	2,674 (11.4%)	1,933 (35.6%)	
HDL-C (mmol/L)	1.39 (1.38, 1.40)	1.38 (1.36, 1.40)	0.3944
LDL-C (mmol/L)	2.96 (2.95, 2.97)	2.89 (2.87, 2.91)	<0.0001
TG (mmol/L)	1.27 (1.26, 1.28)	1.38 (1.35, 1.41)	<0.0001
UA (mg/L)	9.09 (8.90, 9.28)	164.80 (146.15, 183.45)	<0.0001
UCr (mg/dL)	120.86 (119.14, 122.58)	112.68 (110.16, 115.21)	<0.0001
BUN (mmol/L)	12.87 (12.74, 12.99)	17.77 (17.51, 18.04)	<0.0001
TC (mmol/L)	5.05 (5.03, 5.08)	5.03 (4.98, 5.07)	0.3168
SCR (mmol/L)	74.79 (74.44, 75.15)	98.74 (96.82, 100.66)	<0.0001
SUA (mmol/L)	315.72 (314.18, 317.26)	354.73 (351.19, 358.27)	<0.0001
UACR (9mg/g)	7.74 (7.63, 7.85)	170.31 (151.49, 189.14)	<0.0001
eGFR (mL/min/1.73 m^2^)	95.86 (95.38, 96.35)	70.24 (69.17, 71.32)	<0.0001
Vitamin B2 (mg)	2.21 (2.19, 2.24)	1.98 (1.94, 2.02)	<0.0001
Vitamin B6 (mg)	2.15 (2.13, 2.18)	1.90 (1.86, 1.93)	<0.0001
Vitamin B12 (mcg)	5.28 (5.17, 5.38)	4.88 (4.69, 5.08)	0.0003
Calcium (mg)	981.44 (969.98, 992.89)	860.45 (842.00, 878.90)	<0.0001
Magnesium (mg)	307.97 (304.39, 311.55)	272.93 (267.75, 278.12)	<0.0001
Total energy (kcal)	2,138.51 (2,122.04, 2,154.98)	1,878.57 (1,848.43, 1,908.70)	<0.0001
Total fat (g)	82.66 (81.84, 83.47)	73.56 (72.27, 74.85)	<0.0001

CKD: chronic kidney disease; BMI: body mass index; PIR: the ratio of family income to poverty; HDL-C: high-density lipoprotein cholesterol; LDL-C: low-density lipoprotein cholesterol; SUA: serum uric acid; SCR: serum creatinine; BUN: blood urea nitrogen; TC: total cholesterol; TG: serum concentrations of triglycerides; UCr: urinary creatinine; UA: urinary levels of albumin.

Continuous variables are presented as the mean ± standard deviation, and categorical variables showed with *n* (%). *p* < 0.05 means statistically significant.

### 3.2. Association between dietary vitamin B2 intake and CKD

To delineate the association between dietary vitamin B2 exposure and CKD susceptibility, multivariable logistic regression modeling was implemented, with comprehensive outcomes delineated in Table 2. Three hierarchically adjusted analytical frameworks were constructed: model 1 (crude, unadjusted); model 2 (partially adjusted for demographic covariates); and model 3 (fully adjusted for clinical and lifestyle confounders). When riboflavin intake was operationalized as a continuous variable, all models demonstrated statistically significant and robust inverse correlations with CKD prevalence. Upon quartile-based stratification of dietary riboflavin exposure, a significant monotonic inverse relationship with CKD manifestation persisted across all analytical frameworks. Furthermore, the magnitude of nephroprotection exhibited progressive augmentation with ascending quartiles of riboflavin consumption.

**Table 2.** Association between dietary riboflavin (vitamin B2) intake and chronic kidney disease (CKD) prevalence: results from weighted multivariable logistic regression models.

Characteristics	Model 1 OR (95% CI) *p* value	Model 2 OR (95% CI) *p* value	Model 3 OR (95% CI) *p* value
Vitamin B2	0.791 (0.753, 0.831) <0.001	0.869 (0.820, 0.921) <0.001	0.812 (0.686, 0.962) 0.018
Vitamin B2 quartile			
Q1	Reference	Reference	Reference
Q2	0.844 (0.756, 0.941) 0.003	0.855 (0.757, 0.966) 0.012	0.786 (0.627, 0.985) 0.040
Q3	0.667 (0.600, 0.742) <0.001	0.709 (0.620, 0.810) <0.001	0.654 (0.487, 0.877) 0.006
Q4	0.547 (0.483, 0.618) <0.001	0.658 (0.569, 0.762) <0.001	0.573 (0.400, 0.821) 0.003

Model 1: no adjustment. Model 2: adjusted for age, sex,marital status, education level, PIR, and race. Model 3: adjusted for age, sex,marital status, education level, PIR, race, BMI, smoking status, TG, TC, HDL-C, LDL-C, SUA, SCR, BUN, UA, UCr, hypertension, diabetes, vitamin B6, vitamin B12, calcium, total energy, and total fat.

### 3.3. Subgroup analyses and tests for interaction

**Figure 2.**
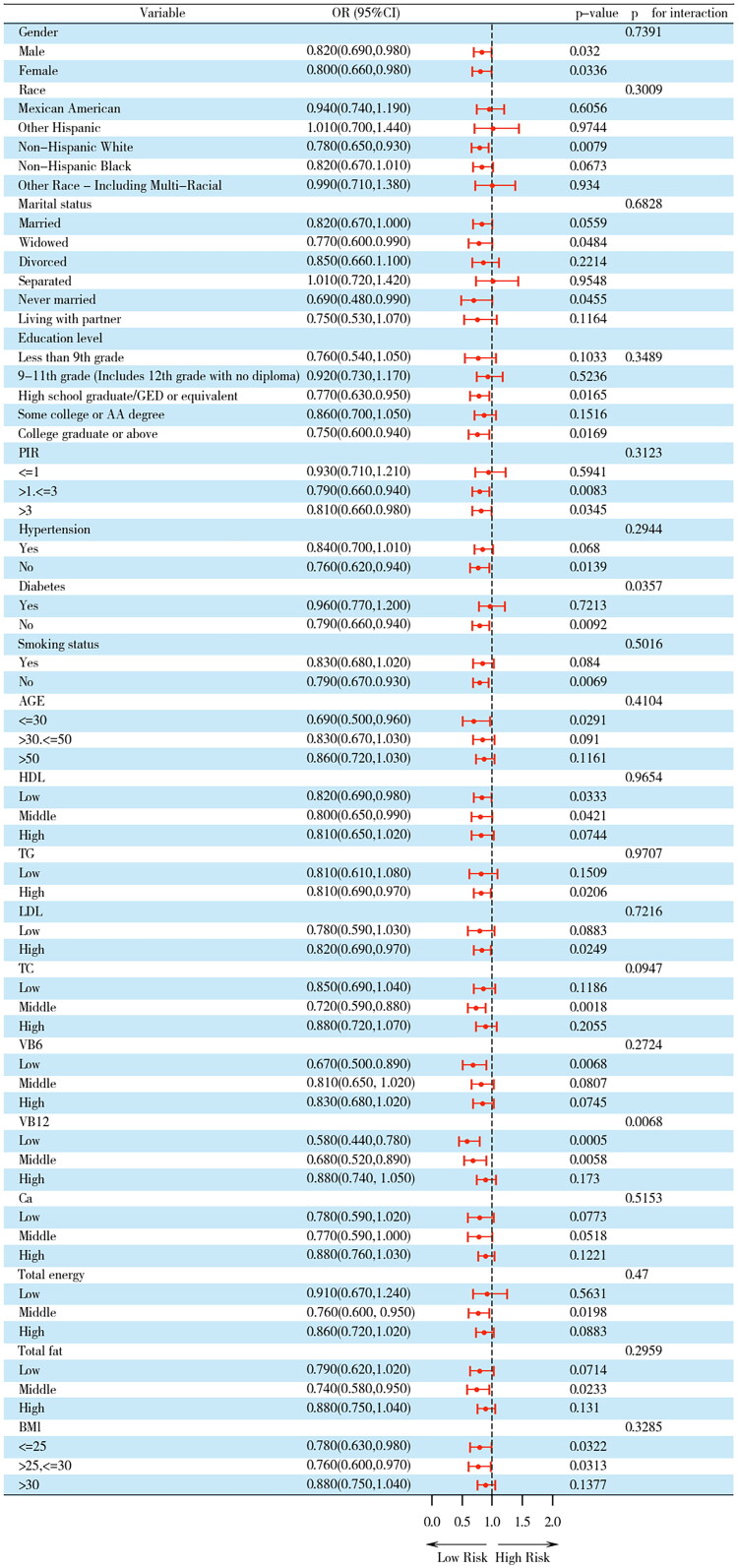
Subgroup analysis. Subgroup analysis and interaction analysis between total dietary vitamin B2 intake and CKD. Subgroup analyses were conducted utilizing a weighted logistic regression model. Interaction analyses were executed employing likelihood-ratio tests.

Stratified subgroup analyses with formal interaction testing were executed to interrogate the homogeneity of the vitamin B2–CKD association across diversified demographic and clinical strata. Interaction was tested by incorporating multiplicative interaction terms (vitamin B2 × subgroup variable) into the logistic regression models, with significance assessed using the likelihood-ratio test. Dendrographic representations meticulously delineate ORs with concomitant 95% CIs characterizing each stratified variable’s influence on CKD risk trajectories. As shown in Figure 2, participants were stratified by age; sex, race, educational level, marital status, PIR, TG, TC, HDL-C, LDL-C, VB6, VB12, Ca, total energy, total fat, smoking status, diabetes, and hypertension. Interaction analyses unveiled significant between-strata divergences in the riboflavin–CKD association among diabetic versus non-diabetic cohorts (*p*-interaction = 0.0357), alongside differential effect magnitudes contingent upon VB12 intake tertiles (*p*-interaction = 0.0068).

### 3.4. Nonlinear relationship and threshold effect analysis between dietary vitamin B2 intake and CKD

**Figure 3.**
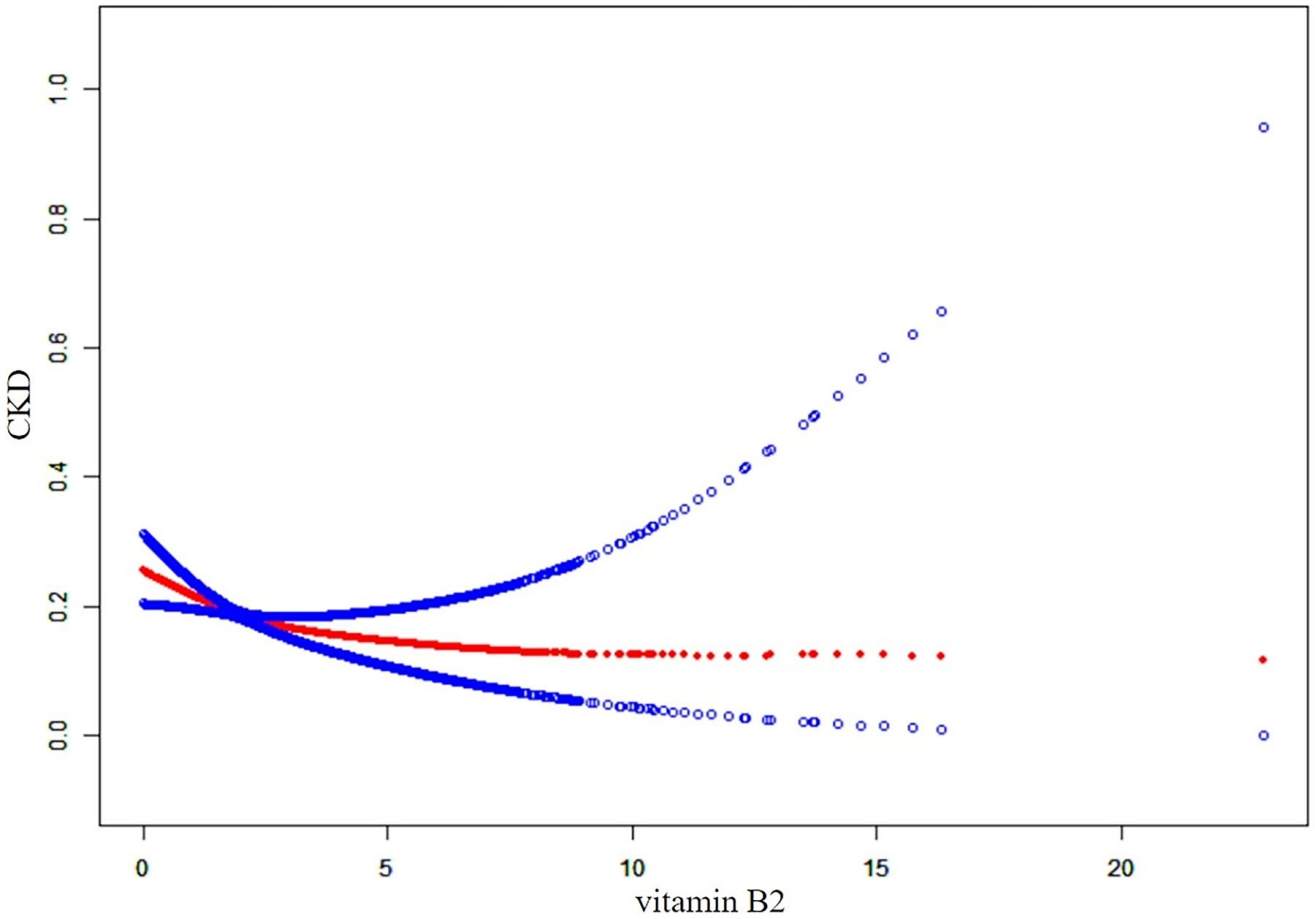
The nonlinear association between dietary vitamin B2 intake and CKD. Solid red lines represent smooth curve fits between variables. The blue band indicates the 95% confidence interval of the fit.

Curve-fitting analysis was employed to examine the inverse relationship between vitamin B2 intake and CKD for curve generation. Figure 3 depicts the observed negative linear correlation between CKD incidence and vitamin B2 consumption, identified as a protective factor against CKD. A nonlinear curve with a significant inflection point at 1.66 mg/d is presented in Table 3 and Figure 3. The association was markedly stronger below this threshold. For vitamin B2 levels below 1.66, a statistically significant inverse association with CKD was noted (OR = 0.67, 95% CI: 0.53–0.85; *p* = 0.0012). However, once vitamin B2 reached 1.66 g, the OR increased slowly until reaching a saturation point of 0.93 (95% CI: 0.83–1.05; *p* = 0.2630).

**Table 3.** Threshold effect analysis of dietary riboflavin (vitamin B2) intake on chronic kidney disease (CKD) prevalence using a two-piecewise linear regression model.

	Adjusted OR (95% CI), *p* value
Vitamin B2	
Inflection point	1.66
Vitamin B2 < 1.66	0.67 (0.53, 0.85) 0.0012
Vitamin B2 ≥ 1.66	0.93 (0.83, 1.05) 0.2630
Log-likelihood ratio	0.012

Linear regression model was used to analyze the threshold effect of dietary vitamin B2 intake on CKD. Adjusting age, sex, marital status, education level, PIR, race, BMI, smoking status, TG, TC, HDL-C, LDL-C, SUA, SCR, BUN, UA, UCr, hypertension, diabetes, vitamin B6, vitamin B12, calcium, total energy, and total fat.

### 3.5. Sensitivity analysis

To assess potential confounding effects of dietary patterns, we performed sensitivity analyses stratified by protein intake levels. As presented in Table 4, the inverse association between vitamin B2 and CKD persisted across all protein intake strata, with a more pronounced protective effect observed in the low protein intake group (OR = 0.881, 95% CI: 0.772–1.005). A statistically significant interaction was identified between vitamin B2 and protein intake (*p*-interaction = 0.003).

**Table 4.** Sensitivity analysis stratified by protein intake levels.

Variable	OR (95% CI)	*p* Value	*p* for interaction
Vitamin B2 (overall)	0.927 (0.849–1.011)	0.091	
Stratified by protein intake			0.003
Low protein intake	0.881 (0.772–1.005)	0.063	
High protein intake	0.951 (0.854–1.058)	0.355	

### 3.6. Potential mechanisms and targets of riboflavin protection in CKD

Intersectional analysis of riboflavin-associated genes (*n* = 105) and CKD-related genes (*n* = 18,128) identified 101 overlapping molecular targets. To reveal the interactions of each protein, a PPI network of shared genes was constructed based on the STRING database, containing 363 edges and 101 nodes. Then, Cytoscape software was used for analysis, and the MCODE plugin was used to identify important modules in the PPI network. The results showed that 43 hub genes in the key cluster were closely connected as important modules.

**Figure 4.**
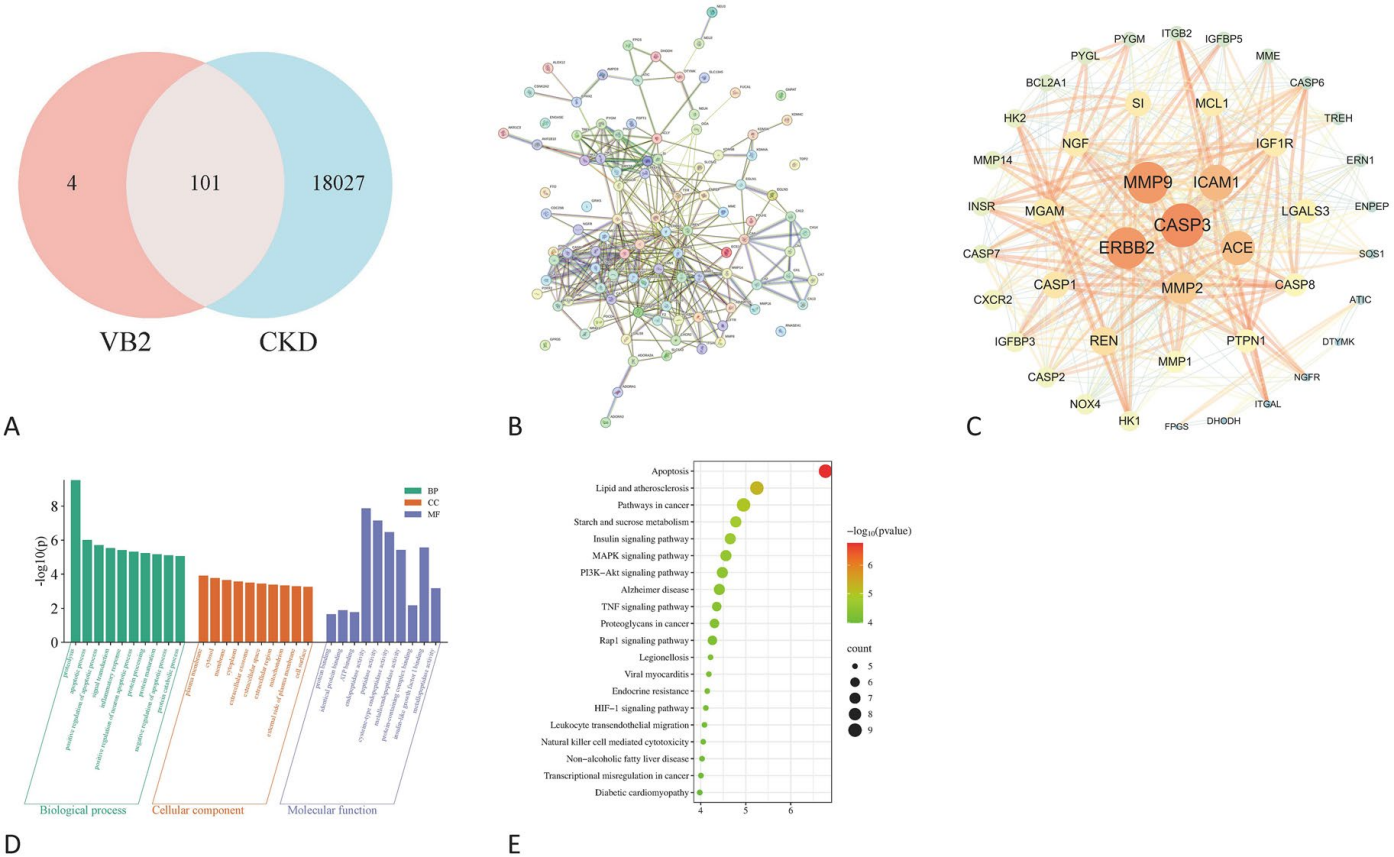
Network pharmacological analysis. Potential targets for improving CKD by (A) VB2, (B) PPI network construction, and (C) hub gene identification. A larger node size indicates a higher degree value, and orange and blue coloring correspond to higher and lower degree values, respectively. (D) GO enrichment analyses for the VB2 protect CKD (*p* < 0.05). (E) The most enriched KEGG pathway associated with VB2 protecting CKD-related target genes.

Through the DAVID database, we found 235 GO terms related to these target genes (*p* < 0.05), distributed across 147 biological process (BP), 31 cellular component (CC), and 57 molecular function (MF). As rendered in Figure 4(A), these target genes manifested predominant overrepresentation within BP annotations – notably encompassing proteolysis, apoptotic process, positive regulation of apoptotic process, signal transduction, and inflammatory response. Substantial enrichment was concurrently observed among CC terms, including plasma membrane, cytosol, membrane, cytoplasm, and extracellular exosome. And MF domains such as protein binding, identical protein binding, ATP binding, endopeptidase activity, and peptidase activity. KEGG pathway enrichment analysis revealed 51 significantly signaling cascades (*p* < 0.05), with preeminent enrichment identified in apoptosis, lipid, and atherosclerosis, pathways in cancer, starch and sucrose metabolism and insulin signaling pathway. Therefore, these pathways may be related to the mechanism by which riboflavin protects against CKD.

### 3.7. Molecular docking

**Figure 5.**
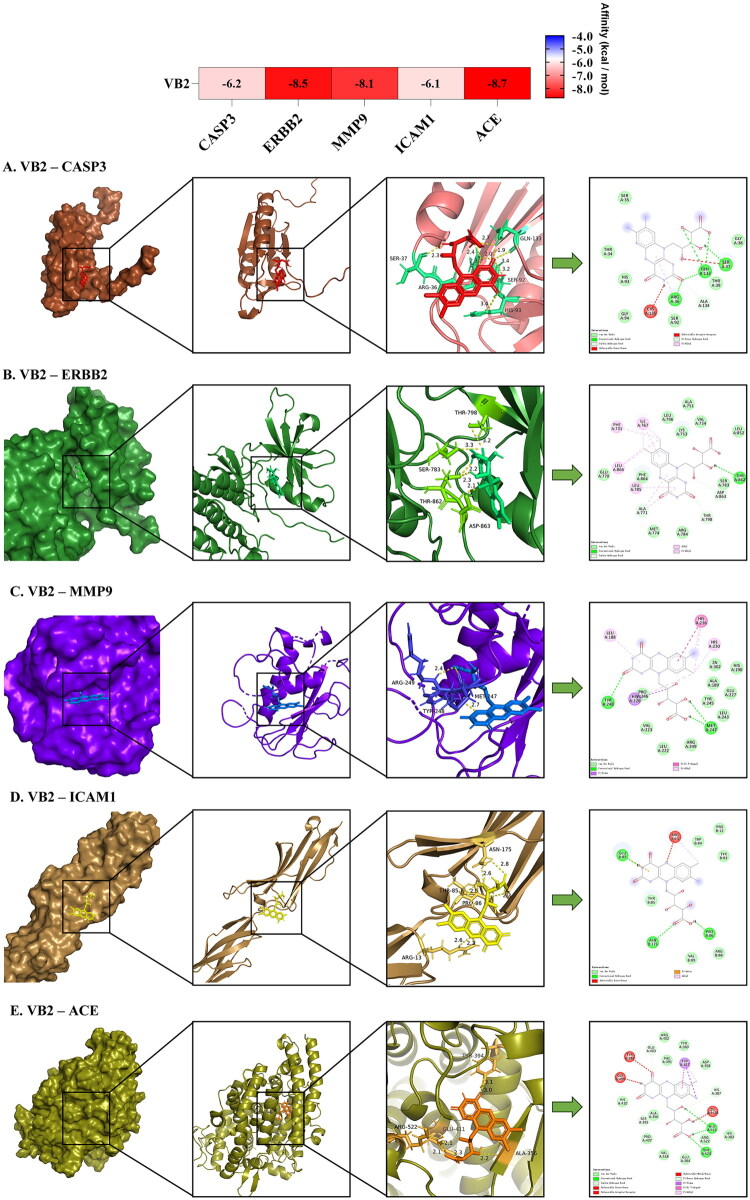
Molecular docking results for VB2 compounds bound to key protein targets, highlighting the complexes with the lowest binding energies. (A) VB2–CASP3; (B) VB2–ERBB2; (C) VB2–MMP9; (D) VB2–ICAM1; (E) VB2–ACE.

Ensuing computational docking analyses were executed between riboflavin and the quintessential core target proteins (CASP3, ERBB2, MMP9, ICAM1, and ACE). Within the computationally generated affinity heatmaps, chromatic gradations toward the red and blue spectra denote diminished and elevated binding free energy values, respectively, reflecting stronger and weaker intermolecular affinities. Binding free energies below the threshold of −5.6 kcal/mol were interpreted as constituting evidence of robust molecular interaction [26]. It is worth noting that most of the screened compounds have a binding affinity to the core molecular target lower than −5.6 kcal/mol. This pharmacological profile implicates riboflavin in exerting renoprotective actions through polypharmacological engagement of multiple pathophysiological targets. Representative binding conformations, characterized by minimal binding free energies and optimal conformational stability within respective compound cohorts, were selected for visualization of stable intermolecular interactions (Figure 5). The specific box coordinates and grid size of the protein–ligand binding site are outlined in Supplementary Table S1.

### 3.8. Molecular dynamics simulation

**Figure 6.**
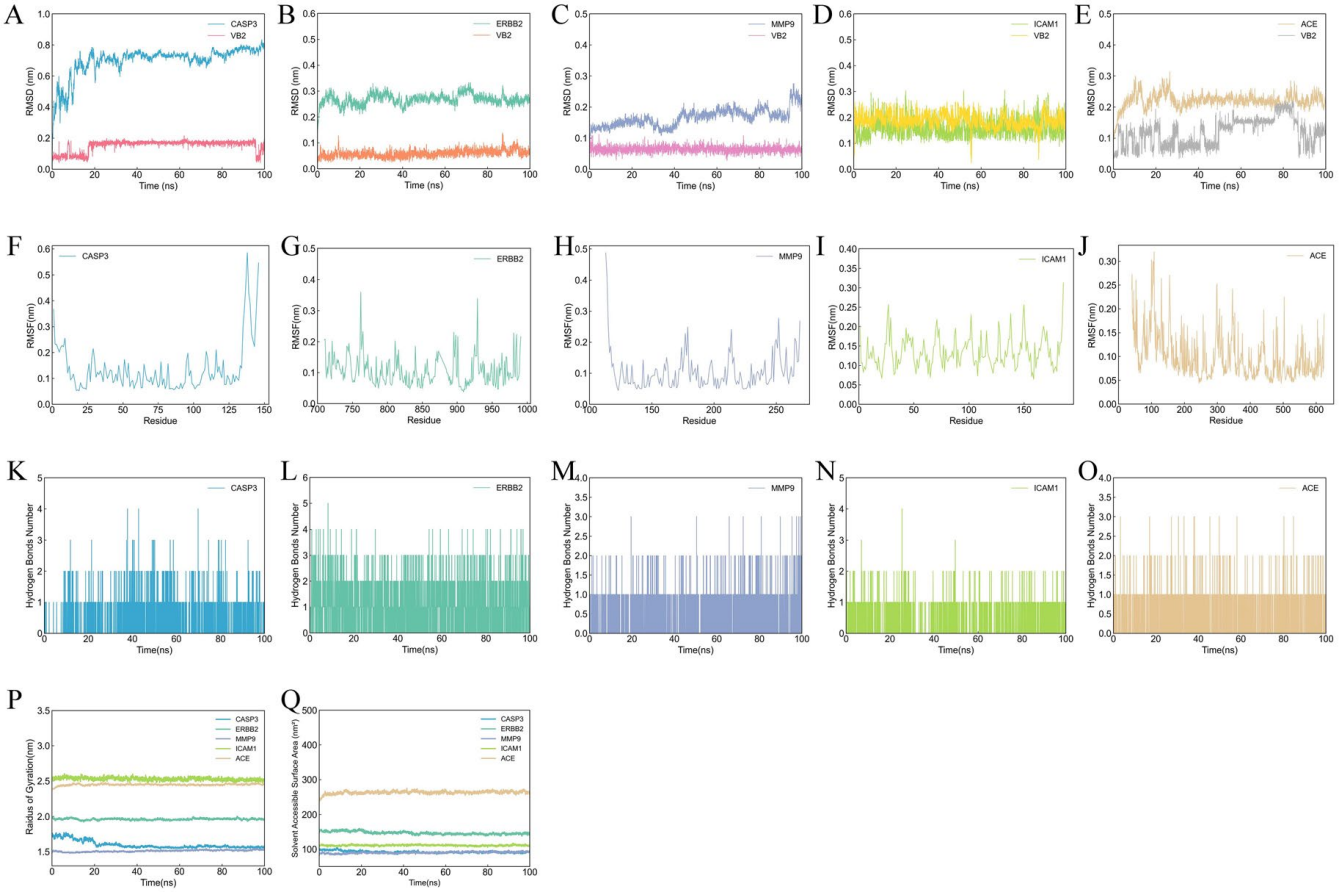
Molecular dynamics simulations. (A–E) The RMSD plot of VB2–CASP3, VB2–ERBB2, VB2–MMP9, VB2–ICAM1, and VB2–ACE. (F–J) The RMSF plot of VB2–CASP3, VB2–ERBB2, VB2–MMP9, VB2–ICAM1, and VB2–ACE. (K–O) The hydrogen bond numbers of VB2–CASP3, VB2–ERBB2, VB2–MMP9, VB2–ICAM1, and VB2–ACE. (P) Rg plots of VB2–CASP3, VB2–ERBB2, VB2–MMP9, VB2–ICAM1, and VB2–ACE. (Q) SASA plots of VB2–CASP3, VB2–ERBB2, VB2–MMP9, VB2–ICAM1, and VB2–ACE.

Compounds exhibiting optimal molecular docking affinities with the five core target proteins were subsequently advanced to 100 ns MD simulations. To undertake a thorough assessment of systemic stability, the root mean square deviation (RMSD) metric was employed, which elucidates conformational dynamics within receptor–ligand complexes. Initial trajectories of the VB2–CASP3, VB2–ERBB2, VB2–MMP9, VB2–ICAM1, and VB2–ACE systems manifested significant RMSD fluctuations. Notably, all complexes achieved high conformational stability beyond the 20 ns simulation window, with RMSD values consistently stabilizing below 0.8 nm – indicating that ligand binding does not significantly alter the protein conformation (Figure 6(A–E)). The elevated RMSD profile observed in the VB2–CASP3 complex may be attributable to the comparatively larger atomic count within the CASP3 macromolecular framework. While diminished RMSD values denote structural equilibrium, heightened deviations correlate with conformational instability [27]. Collectively, these findings demonstrate robust stability across all simulated complexes under designated conditions.

To elucidate the influence of amino acids on the binding affinity and conformational stability of target proteins, and to quantitatively assess protein flexibility, MD simulations were conducted utilizing the root mean square fluctuation (RMSF) metric. The findings revealed that the mean RMSF values for the VB2–CASP3, VB2–ERBB2, VB2–MMP9, VB2–ICAM1, and VB2–ACE complexes consistently remained below the threshold of 0.2 nm (Figure 6(F–J)). The low RMSF values indicate minimal movement of the two complexes during the simulations, which suggests that the binding modes are stable with minimal shape changes [28]. Notably, the N-terminal domain of the VB2–MMP9 complex exhibited elevated RMSF values, potentially attributable to the presence of densely packed secondary structural elements, exemplified by α-helices and β-sheets. Conversely, lower RMSF values may signify the absence of corresponding structures within the complex. Hydrogen bonding interactions constitute pivotal regulators of protein–ligand binding affinity and serve as fundamental determinants governing molecular interactions. Our analyses demonstrate that the population of interfacial hydrogen bonds within the majority of temporal sampling windows for these five complexes exhibited temporal variations, ranging from one to four persistent interactions (Figure 6(K–O)).

The radius of gyration (Rg) serves as a pivotal metric for assessing the stability of ligand binding within protein complexes. Computation of Rg facilitates insights into the tertiary structure and conformational dynamics of the protein. Our analyses reveal markedly reduced Rg values for CASP3, ERBB2, MMP9, ICAM1, and ACE. The comparatively elevated Rg observed for ICAM1 is potentially attributable to the atomic count of its cognate macromolecules. Collectively, these diminished Rg values signify the adoption of stable and compact structural conformations by these proteins (Figure 6(P)). Solvent-accessible surface area (SASA), quantified based on the ratio of the protein’s total surface area to its energy, provides valuable information regarding the spatial distribution of solvent molecules surrounding the protein structure [29]. Notably, the decreased SASA values exhibited by CASP3, ERBB2, MMP9, ICAM1, and ACE suggest increased stability within their corresponding protein–ligand complexes (Figure 6(Q)).

The molecular mechanics Poisson–Boltzmann surface area (MM/PBSA) methodology represents an extensively utilized computational approach for elucidating the binding free energies governing the interactions between small molecules and their cognate biomolecular targets [30]. Within the current study, rigorous MD simulations were utilized to assess the structural stability of ligand–receptor complexes across five distinct systems: namely, CASP3, ERBB2, MMP9, ICAM1, and ACE. Utilizing the terminal 10 ns trajectories extracted from these demonstrably stable complexes, comprehensive calculations of binding free energies were executed, accompanied by detailed residue-level energy decomposition analyses. The computed mean binding free energy for the CASP3 complex was determined to be −152.31 kJ/mol, while corresponding values for ERBB2 and MMP9 complexes were established at −237.76 kJ/mol and −212.85 kJ/mol, respectively. Similarly, the ICAM1 complex exhibited an average binding free energy of −106.46 kJ/mol, and the ACE complex yielded a value of −191.19 kJ/mol (Table 5). Collectively, these computational findings robustly indicate that all five ligand–receptor complexes manifest pronounced stability, as unequivocally corroborated by their respective strong binding affinities.

**Table 5.** Binding free energies of complexes in kJ/mol.

Complexes	Gbind (±SEM)	Emm (±SEM)	Epb (±SEM)	Esa (±SEM)	Evdw (±SEM)
VB2–CASP3	−152.31 ± 14.26	−181.86 ± 11.02	50.66 ± 7.74	−21.10 ± 1.36	−181.86 ± 11.02
VB2–ERBB2	−237.76 ± 2.94	−265.65 ± 4.86	50.86 ± 3.94	−22.98 ± 0.36	−265.65 ± 4.86
VB2–MMP9	−212.85 ± 10.81	−228.15 ± 8.94	37.79 ± 5.78	−22.49 ± 0.19	−228.15 ± 8.94
VB2–ICAM1	−106.46 ± 16.55	−131.48 ± 16.58	38.67 ± 10.26	−13.65 ± 1.69	−131.48 ± 16.58
VB2–ACE	−191.19 ± 5.64	−188.98 ± 5.75	47.98 ± 6.71	−25.91 ± 0.49	−188.98 ± 5.75

## 4. Discussion

### 4.1. Summary of main findings

**Figure 7.**
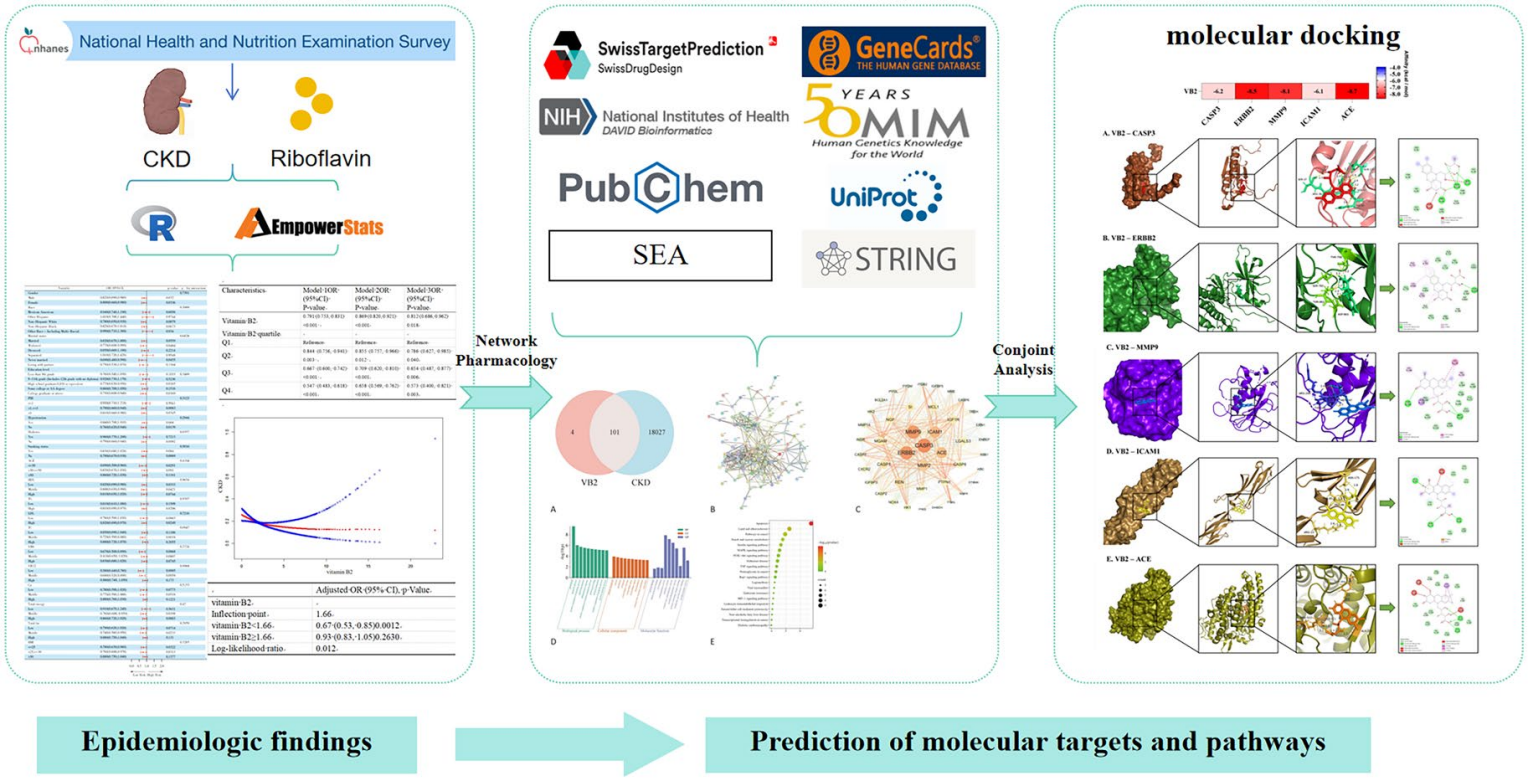
Proposed conceptual framework linking riboflavin intake to reduced risk of CKD. This schematic illustrates the hypothesized pathway from epidemiological observations on riboflavin consumption to the molecular mechanisms conferring nephroprotection.

Leveraging data from the NHANES 2005–2018 cycles, this investigation employed weighted multivariate logistic regression analyses, complemented by interaction assessments, stratified subgroup examinations, and nonlinear curve-fitting methodologies, to elucidate the relationship between dietary vitamin B2 intake and CKD risk. Our analyses revealed a statistically significant nonlinear relationship (*p* = 0.0012) wherein riboflavin intake demonstrated enhanced renoprotective effects below an inflection point of 1.66 mg/day. Notably, this inverse association manifested greater magnitude among non-diabetic individuals, those with lower VB12 and B6 intake, and nonsmokers, as evidenced through rigorous subgroup and interaction analyses. Furthermore, network pharmacology interrogation identified CASP3, ERBB2, MMP9, ICAM1, and ACE as pivotal molecular targets of riboflavin. Mechanistic exploration suggests riboflavin exerts nephroprotective effects potentially via modulation of apoptosis, lipid and atherosclerosis, pathways in cancer, starch and sucrose metabolism, and insulin signaling pathways. Subsequent MD simulations validated the structural stability of ligand target complexes comprising these pivotal proteins. A schematic summary integrating these epidemiological findings with the proposed molecular targets and pathways is presented in Figure 7, illustrating the conceptual framework linking riboflavin intake to CKD risk reduction.

### 4.2. Supporting preclinical and clinical evidence

Supporting preclinical and clinical evidence corroborates our mechanistic findings, demonstrating an inverse correlation between riboflavin intake and CKD pathogenesis. In a diabetic murine model, Alam et al. showed that riboflavin supplementation alleviates renal oxidative stress and genomic instability while enhancing uremic toxin clearance, boosting antioxidant capacity, and preserving renal histoarchitectural integrity collectively attenuating diabetic nephropathy progression [31]. Riboflavin administration facilitates functional recovery of glutathione reductase (GR), the FAD-dependent enzyme crucial for redox homeostasis in renal tissue [32,33]. Subsequent studies confirmed riboflavin-mediated reduction in renal lipid peroxidation and protein carbonylation, along with restored glutathione S-transferase activity [34]. Epidemiologically, Ren et al. found that riboflavin insufficiency significantly increases all-cause mortality risk in CKD patients [35]. This translational evidence spanning molecular pathways to clinical outcomes supports riboflavin repletion as a promising intervention for CKD mitigation through its dual cardiorenal protective properties.

### 4.3. Comparison with other micronutrient studies in CKD

While the roles of micronutrients in CKD have been extensively investigated, riboflavin demonstrates distinct characteristics compared to other vitamins. For instance, vitamin D primarily associates with mineral and bone disorder (CKD-MBD), manifesting as skeletal abnormalities, bone pain, and increased fracture risk [36]. B-complex vitamins, including B6, B12, and folate, have been mainly studied for their involvement in homocysteine metabolism; although supplementation reduces homocysteine levels, clinical benefits for cardiovascular outcomes remain uncertain [37]. Vitamins C and E function as antioxidants with potential for mitigating oxidative stress, yet vitamin C carries risks of oxalate accumulation [38], while vitamin E has not demonstrated consistent renoprotective effects in interventional trials [39,40].

However, the underlying mechanism(s) of riboflavin in CKD remain incompletely understood. Compared to the vitamins mentioned above, riboflavin’s actions concentrate more fundamentally on cellular energy metabolism and redox homeostasis, particularly through its crucial involvement in mitochondrial function and apoptosis regulation. This unique mechanistic profile positions riboflavin as having distinctive potential for early-stage CKD intervention. A systematic comparison of these vitamins regarding their primary functions, mechanisms of action, and evidence strength in CKD is presented in Supplementary Table S2.

### 4.4. Mechanistic insights into riboflavin’s renoprotective effects

Our findings delineate CASP3, ERBB2, MMP9, ICAM1, and ACE as pivotal molecular targets through which riboflavin exerts nephroprotection. The core signaling pathways implicated in riboflavin-mediated CKD prevention converge on apoptosis, lipid metabolism and atherosclerosis, oncogenic pathways, starch/sucrose metabolism, and insulin signaling. To systematically synthesize these interactions, a comprehensive mapping of riboflavin’s proposed functions to these molecular targets and pathways is provided in Supplementary Table S3.

Tubular epithelial cell (TEC) apoptosis constitutes a fundamental pathogenic cascade in CKD progression, wherein TEC senescence and dysfunction exacerbate renal functional deterioration [41,42]. Riboflavin deficiency potentiates this cascade through endoplasmic reticulum stress amplification: upregulating pro-apoptotic mediators (e.g., CHOP, caspase-12) while suppressing anti-apoptotic factors (e.g., Bcl-2), culminating in TEC demise and tubular atrophy [43].

Concurrently, diminished FAD/FMN bioavailability instigates dual pathophysiological derangements – redox imbalance and lipotoxic accrual. The former arises from FAD-dependent GR inactivation, depressing glutathione/glutathione disulfide ratios [44,45], and compromising cellular antioxidant defenses, thereby inducing ROS surge [46,47], However, oxidized low-density lipoprotein (ox-LDL) is produced by ROS through oxidative modification of LDL [48], and a large number of studies have shown that Ox-LDL is a lipid with strong pro-atherogenic effect [49,50]; the latter emanates from impaired FAD-dependent β-oxidation (e.g., acyl-CoA dehydrogenase dysfunction) [51]. Ox-LDL can induce endoplasmic reticulum stress, activate unfolded protein response (UPR) [52], triggering endothelial apoptosis and vascular barrier disruption [53,54], that potentiate inflammatory infiltration and atherogenesis. Within the CKD milieu, this reciprocally reinforcing circuit – tubulointerstitial injury from TEC apoptosis and ox-LDL-driven microangiopathy – concertedly accelerates renal functional decline and cardiovascular events [3,55,56].

It is important to emphasize that the computational docking and MD simulations conducted here are hypothesis-generating; they provide a robust mechanistic premise that warrants future experimental validation in cellular and animal models of CKD.

### 4.5. Subgroup analyses and precision nutrition implications

Subgroup analyses revealed significant interactions, indicating that the inverse association between vitamin B2 intake and CKD was more pronounced in nondiabetic individuals, those with lower dietary VB12/VB6 intake, and never-smokers. Substantial evidence identifies diabetes as a principal etiological factor for CKD [57], wherein renal microvascular complications precipitate diabetic kidney disease (DKD) often characterized by incipient mitochondrial dysfunction. Vitamin B2, functioning as a precursor to flavin coenzymes (FAD/FMN), may confer protection against non-diabetes-related CKD risk factors through antioxidative mechanisms (e.g., GR support) and mitochondrial bioenergetic enhancement [13].

Vitamin B12 serves as an essential cofactor for methionine synthase (MTR), catalyzing homocysteine-to-methionine conversion within one-carbon metabolism [58,59]. Vitamin B12 insufficiency impairs MTR activity, resulting in homocysteine accumulation and consequent hyperhomocysteinemia [60,61] – a well-established nontraditional risk factor for adverse CKD outcomes. Vitamin B6 deficiency similarly reduces cystathionine β-synthase (CBS) activity, diminishing cystathionine biosynthesis [62].

Tobacco exposure elevates CKD risk [63], via endothelial toxicity from nicotine, heavy metals (e.g., cadmium) [64], and free radical-mediated oxidative stress/inflammation [65]. In never-smokers, vitamin B2’s dual capacities in redox homeostasis and metabolic regulation may more effectively modulate CKD pathogenesis. Consequently, targeted preventive strategies for high-risk cohorts could attenuate CKD incidence and progression trajectories.

This finding underscores the heterogeneous nature of this association and strongly suggests that a one-size-fits-all nutritional intervention strategy may be insufficient for optimizing outcomes in the CKD population. Instead, our data support the potential value of precision nutrition – an individualized approach utilizing person-specific data such as genetics, metabolic profiles, and comorbidities – in the prevention and management of CKD [66]. Current mainstream nutritional management, as recommended by KDIGO guidelines, is primarily based on population-level evidence and provides a generalized framework for CKD patients, emphasizing protein, phosphorus, potassium, and sodium restriction while ensuring adequate energy intake [67]. Although these recommendations are essential, they may not fully address the unique physiological and metabolic needs of individual patients. In this context, our study provides preliminary evidence for incorporating riboflavin into precision nutrition strategies for CKD. Riboflavin requirements or metabolic efficiency may be influenced by genetic background, comorbidities, the status of other nutrients, or lifestyle factors. Consequently, future nutritional assessments for CKD patients should extend beyond traditional parameters to include the identification of high-risk subgroups who may benefit from riboflavin optimization. With advancing precision nutrition tools such as nutrigenetics, metabolomics, and microbiome analytics, more refined stratification could enable truly individualized riboflavin supplementation regimens. This approach would complement KDIGO guidelines, forming a more comprehensive and efficient new paradigm for nutritional management in CKD.

### 4.6. Strengths and limitations

Our investigation possesses several salient strengths. First, the study leverages a nationally representative population cohort, furnishing robust statistical power through sufficient sample sizing and weighted analyses to bolster extrapolative validity. Second, we integrated epidemiological findings with computational biology, utilizing network pharmacology, molecular docking, and dynamics simulations to propose a mechanistic basis for riboflavin’s renoprotective effects, thereby bridging observational correlation with molecular-level hypotheses.

Notwithstanding these contributions, several limitations warrant acknowledgment. Primarily, the quantification of dietary riboflavin intake was based on two 24-h recall interviews, a method susceptible to recall bias that may compromise the accuracy of intake estimates, despite its common use in large-scale nutritional epidemiology due to practical constraints in assessing habitual diet. Second, the cross-sectional nature of the NHANES data prevents causal inference, allowing only the identification of associative relationships between riboflavin consumption and the prevalence of CKD. Third, the subgroup and interaction analyses, while informative, were exploratory in nature. The *p* values for interaction were not adjusted for multiple comparisons. Therefore, these findings should be interpreted as hypothesis-generating and require confirmation in future studies. Fourth, our computer program exhibits limitations in task execution. Molecular docking and MD simulations were not validated against known positive control ligands. The riboflavin quantities employed in computational testing represent theoretical values designed to elucidate binding mechanisms, potentially differing from concentrations in human blood (typically far lower). While simulation results offer valuable insights into complex binding strength and stability, further experimentation under conditions approximating *in vivo* environments remains necessary to validate these findings’ applicability to biological systems. Lastly, this study is based on a population of adults in the United States, but there are differences in ethnicity, economic status, lifestyle, and other factors among CKD patients in different countries. It emphasizes the necessity of longitudinal studies to ascertain potential causal links, with further clinical intervention studies across diverse populations and regions being requisite to corroborate these observations and enhance the generalizability of the findings.

### 4.7. Translational implications

This integrated analysis not only establishes a link between dietary riboflavin intake and renal health but also identifies potential targets and pathways underlying this relationship. These findings provide a foundation for developing precision nutrition strategies for CKD, particularly in high-risk populations who may benefit from riboflavin optimization. Furthermore, the candidate targets and pathways identified – especially those involved in apoptosis and metabolic regulation offer a theoretical and experimental basis for future development of novel nutritional formulations or targeted pharmacological interventions to slow CKD progression. Compared to other vitamins, research on riboflavin in the context of CKD remains comparatively underdeveloped, underscoring the need to promote its inclusion in future clinical intervention trials.

### 4.8. Implications for clinical and basic science research

This study pioneers the inclusion of riboflavin as a potential candidate molecule for nutritional intervention in CKD, proposing a multi-target mechanistic framework for its action (Supplementary Table S3). At the clinical level, it is recommended to implement riboflavin intake screening in high-risk CKD populations and to design randomized controlled trials to validate its preventive efficacy. For basic research, the targets and pathway hypotheses presented here offer a direction for subsequent cellular and animal experiments, thereby facilitating the elucidation of the specific mechanisms underlying riboflavin’s role in renal protection.

## 5. Conclusions

In conclusion, this study reveals a significant inverse association between dietary riboflavin intake and CKD prevalence among U.S. adults, characterized by a nonlinear relationship with enhanced protective effects below an intake threshold of 1.66 mg/day. Integrated network pharmacology and molecular docking identified CASP3, ERBB2, MMP9, ICAM1, and ACE as key targets for riboflavin, underscoring its potential role in modulating apoptosis, lipid metabolism, and insulin signaling for nephroprotection. These findings not only reinforce the potential role of riboflavin as a dietary strategy for CKD prevention but also provide a mechanistic foundation for future research. Further experimental studies and randomized controlled trials are necessary to validate these observations and explore the translational potential of riboflavin supplementation in CKD management.

## Supplementary Material

Supplementary Table S3.docx

Supplementary Table S2.docx

Supplementary Table S4.docx

STROBE Statement.docx

Supplementary Table S1.docx

## Data Availability

The data that support the findings of this study are available in the National Health and Nutrition Examination Survey (NHANES) at https://www.cdc.gov/nchs/nhanes/. These data were derived from the following resource available in the public domain: https://wwwn.cdc.gov/nchs/nhanes/.
