# Supplementary material for: Riboflavin intake and kidney health: population evidence and mechanistic insights from NHANES and molecular docking analyses
Source: Ren Fail. 2026 Jan 25;48(1):2611520. doi: 10.1080/0886022X.2025.2611520 (PMC12836406; doi:10.1080/0886022X.2025.2611520)
Supplement: Supplementary Table S3.docx [file IRNF_A_2611520_SM9513.docx]

| The presumed functions of vitamin B2 | Relevant key molecular targets | Core pathways/processes involved | The potential pathophysiological significance in CKD |
| --- | --- | --- | --- |
| Inhibition of apoptosis and oxidative stress | CASP3 | Apoptosis | Reduce tubular epithelial cell death and protect renal function[1]. |
| Regulating Blood Pressure and Fibrosis | ACE | Renin-angiotensin system | Inhibits the production of angiotensin II, thereby improving hypertension and renal fibrosis[2]. |
| Regulating Blood Pressure and Fibrosis | MMP9, ICAM1 | Lipid and atherosclerosis | Reduce extracellular matrix degradation and leukocyte infiltration, thereby delaying vascular inflammation and glomerulosclerosis.[3] |
| Regulate cell growth and metabolism | ERBB2 | Insulin signaling pathway/Pathways in cancer | It may indirectly influence renal pathological alterations by affecting cellular proliferation and metabolic homeostasis[4]. |

Supplementary Table S3. Summary of proposed mechanisms: mapping riboflavin's potential functions, molecular targets, and pathway relevance to CKD.

## Reference

1. Yamamoto K, Tomita N, Yoshimura S, Nakagami H, Taniyama Y, Yamasaki K, et al. Hypoxia-induced renal epithelial cell death through caspase-dependent pathway: role of Bcl-2, Bcl-xL and Bax in tubular injury. Int J Mol Med. 2004;14(4):633-40.

2. Ford Versypt AN, Harrell GK, McPeak AN. A pharmacokinetic/pharmacodynamic model of ACE inhibition of the renin-angiotensin system for normal and impaired renal function. Computers & Chemical Engineering. 2017;104:311-22.

3. Panagopoulos A, Samant S, Bakhos JJ, Liu M, Khan B, Makadia J, et al. Triggering receptor expressed on myeloid cells-1 (TREM-1) inhibition in atherosclerosis. Pharmacology & Therapeutics. 2022;238:108182.

4. Kuczkowski A, Brinkkoetter PT. Metabolism and homeostasis in the kidney: metabolic regulation through insulin signaling in the kidney. Cell Tissue Res. 2017;369(1):199-210.
