# Supplementary material for: Riboflavin intake and kidney health: population evidence and mechanistic insights from NHANES and molecular docking analyses
Source: Ren Fail. 2026 Jan 25;48(1):2611520. doi: 10.1080/0886022X.2025.2611520 (PMC12836406; doi:10.1080/0886022X.2025.2611520)
Supplement: Supplementary Table S2.docx [file IRNF_A_2611520_SM9512.docx]

| Micronutrien | Primary Functions and Mechanisms | Associations and Evidence in CKD | Contrast with Riboflavin in This Study |
| --- | --- | --- | --- |
| Vitamin D | Calcium-Phosphate Homeostasis: Active form (1,25-(OH)₂D) enhances intestinal calcium absorption and regulates PTH[1]. RAAS Inhibition:Suppresses renin-angiotensin-aldosterone system activation[2]. Anti-inflammatory & Antioxidant: Modulates immune response and reduces oxidative stress. Endothelial Function: Improves vasodilation and promotes vascular repair[3]. | Deficiency is highly prevalent. Strongly associated with CKD-MBD[4], increased cardiovascular risk[5], cognitive impairment[6], and Major Adverse Kidney Events (MAKEs)[7]. Supplementation is a cornerstone of standard care. | Riboflavin does not primarily target mineral metabolism. Its role is centered on fundamental cellular energy metabolism and redox homeostasis, operating at a more basic level of cellular function. |
| Folate / B12 | Homocysteine Metabolism:Key coenzymes in homocysteine remethylation to methionine[8]. DNA Synthesis: Essential for DNA synthesis, repair and methylation processes. | Deficiency leads to hyperhomocysteinemia[9], anemia[10], neurological complications[11], and immune dysfunction[12]. Supplementation lowers homocysteine levels but benefits on hard endpoints remain controversial. | Riboflavin is not directly involved in one-carbon metabolism. Its protection targets more fundamental cellular processes including mitochondrial function and apoptosis regulation. |
| Vitamin B6 | Amino Acid Metabolism: PLP participates in transamination, decarboxylation, and racemization reactions[13]. Heme Synthesis: Cofactor for rate-limiting enzyme in heme biosynthesis[14]. Homocysteine Metabolism: Participates in homocysteine transsulfuration[15]. Immune Regulation: Essential for proper immune function. | Deficiency may relate to anemia, neurological symptom[16]s, and hyperhomocysteinemia[17]. Combined use with folate/B₁₂ can help lower Hcy. | Riboflavin (B₂) is a prerequisite for PLP synthesis. While B₆ functions in specific metabolic pathways, riboflavin serves as a more fundamental cofactor for core cellular energetics and redox balance. |
| Vitamin C | Antioxidant Defense: Potent water-soluble antioxidant that scavenges ROS[18]. Endothelial Protection: Promotes NO production and improves vascular function[19]. Anti-inflammatory: Modulates inflammatory cytokine production[20]. | Levels may be low due to dietary restrictions and dialysate losses. High-dose supplementation carries risk of oxalate deposition, limiting its therapeutic use. Deficiency exacerbates oxidative stress, inflammation, and infection risk[21]. | Riboflavin supports the endogenous antioxidant system (e.g., glutathione recycling) rather than acting directly as a radical scavenger, providing a more sustainable antioxidant strategy. |
| Vitamin E | Lipid-Soluble Antioxidant: Protects membranes from lipid peroxidation[22]. Anti-inflammatory: Modulates inflammatory responses[23]. | Potential for improving oxidative stress and inflammation has been explored, but evidence from interventional studies is weak and inconsistent. Not routinely recommended in CKD management. | Riboflavin's role encompasses both energy production (FAD in ETC) and antioxidant defense (GR activity), positioning it as a central metabolic regulator rather than solely an antioxidant. |
| Riboflavin (B2) | Precursor of FAD and FMN; core roles in: 1. Redox metabolism (e.g., glutathione reductase, GR) 2. Mitochondrial energy metabolism (e.g., fatty acid β-oxidation) 3. Key pathways such as apoptosis[24]. | This study and previous work suggest that its deficiency is independently associated with oxidative stress, mitochondrial dysfunction, and increased CKD risk . Emerging evidence supports its potential in CKD prevention. | Focus of this study: Mechanistically distinct from other vitamins; directly supplies essential cofactors for fundamental cellular metabolism and survival pathways. |

Supplementary Table S2. Comparison of Mechanisms of Action and Evidence for Major Micronutrients in CKD.

## Reference

1. Levin A, Li YC. Vitamin D and its analogues: do they protect against cardiovascular disease in patients with kidney disease? Kidney Int. 2005;68(5):1973-81.

2. Kaur G, Singh J, Kumar J. Vitamin D and cardiovascular disease in chronic kidney disease. Pediatr Nephrol. 2019;34(12):2509-22.

3. Apetrii M, Covic A. Vitamin D and Endothelial Function in Chronic Kidney Disease. In: Ureña Torres PA, Cozzolino M, Vervloet MG, editors. Vitamin D in Chronic Kidney Disease. Cham: Springer International Publishing; 2016. p. 343-59.

4. Nigwekar SU, Tamez H, Thadhani RI. Vitamin D and chronic kidney disease-mineral bone disease (CKD-MBD). Bonekey Rep. 2014;3:498.

5. Saleem A, Padakanti SS, Hajjaj M, Akram MS, Siddenthi SM, Kumari V, et al. Effects of Vitamin D Supplementation on Cardiovascular Outcomes in Chronic Kidney Disease Patients: A Systematic Review and Meta-Analysis. Cureus. 2025;17(7):e87378.

6. Cheng Z, Lin J, Qian Q. Role of Vitamin D in Cognitive Function in Chronic Kidney Disease. Nutrients. 2016;8(5).

7. Lin YM, Kao CL, Hung KC, Liu TH, Yu T, Liu MY, et al. Major adverse kidney events among chronic kidney disease patients with vitamin D deficiency. Front Nutr. 2025;12:1650514.

8. Courseault J, Kingry C, Morrison V, Edstrom C, Morrell K, Jaubert L, et al. Folate-dependent hypermobility syndrome: A proposed mechanism and diagnosis. Heliyon. 2023;9(4):e15387.

9. Paliienko IA, Karpenko OV, Krasiuk I, Kravchuk AD, Mykolaienko YV, Rudenko OA. Hyperhomocysteinemia is a risk factor for vascular complications in patients with chronic kidney disease. KIDNEYS. 2024.

10. Wu HHL, Wang AY. Vitamin B12 and chronic kidney disease. Vitam Horm. 2022;119:325-53.

11. Ramprasad K, Siddappa MN. A comparative study of total vitamin B12 and active B12 (holotranscobalamin) in patients with chronic kidney disease. International Journal of Clinical Biochemistry and Research. 2024.

12. Adhikari PM, Chowta MN, Ramapuram JT, Rao SB, Udupa K, Acharya SD. Effect of Vitamin B12 and folic acid supplementation on neuropsychiatric symptoms and immune response in HIV-positive patients. J Neurosci Rural Pract. 2016;7(3):362-7.

13. Suzuki Y, Matsuzawa R, Hoshi K, Koh YM, Yamamoto S, Harada M, et al. Comparative Analysis of Simplified, Objective Nutrition-Associated Markers in Patients Undergoing Hemodialysis. J Ren Nutr. 2022;32(4):458-68.

14. Searcy K, Rainwater S, Jeroudi M, Baliga R. Erythropoietin-stimulating agent-resistant vitamin B(6) deficiency anemia in a pediatric patient on hemodialysis. Pediatr Nephrol. 2021;36(2):473-6.

15. Carracedo J, Alique M, Vida C, Bodega G, Ceprián N, Morales E, et al. Mechanisms of Cardiovascular Disorders in Patients With Chronic Kidney Disease: A Process Related to Accelerated Senescence. Front Cell Dev Biol. 2020;8:185.

16. Tong Y. Seizures caused by pyridoxine (vitamin B6) deficiency in adults: A case report and literature review. Intractable Rare Dis Res. 2014;3(2):52-6.

17. Chen W, Feng J, Ji P, Liu Y, Wan H, Zhang J. Association of hyperhomocysteinemia and chronic kidney disease in the general population: a systematic review and meta-analysis. BMC Nephrology. 2023;24(1):247.

18. Sato A, Kondo Y, Ishigami A. The evidence to date: implications of l-ascorbic acid in the pathophysiology of aging. J Physiol Sci. 2024;74(1):29.

19. Takahashi N, Morimoto S, Okigaki M, Seo M, Someya K, Morita T, et al. Decreased plasma level of vitamin C in chronic kidney disease: comparison between diabetic and non-diabetic patients. Nephrol Dial Transplant. 2011;26(4):1252-7.

20. Wang C, Zhao J, Zhou Q, Li J. Serum vitamin C levels and their correlation with chronic kidney disease in adults: a nationwide study. Ren Fail. 2024;46(1):2298079.

21. Hongsawong N, Chawprang N, Kittisakmontri K, Vittayananan P, Srisuwan K, Chartapisak W. Vitamin C deficiency and impact of vitamin C administration among pediatric patients with advanced chronic kidney disease. Pediatr Nephrol. 2021;36(2):397-408.

22. Fryer MJ. Vitamin E as a protective antioxidant in progressive renal failure. Nephrology. 2000;5.

23. Fang J, Xie S, Chen Z, Wang F, Chen K, Zuo Z, et al. Protective Effect of Vitamin E on Cadmium-Induced Renal Oxidative Damage and Apoptosis in Rats. Biol Trace Elem Res. 2021;199(12):4675-87.

24. Henriques BJ, Olsen RK, Bross P, Gomes CM. Emerging roles for riboflavin in functional rescue of mitochondrial β-oxidation flavoenzymes. Curr Med Chem. 2010;17(32):3842-54.
