# Supplementary material for: Riboflavin intake and kidney health: population evidence and mechanistic insights from NHANES and molecular docking analyses
Source: Ren Fail. 2026 Jan 25;48(1):2611520. doi: 10.1080/0886022X.2025.2611520 (PMC12836406; doi:10.1080/0886022X.2025.2611520)
Supplement: Supplementary Table S4.docx [file IRNF_A_2611520_SM9511.docx]

| Model | Level of Adjustment | Covariates Included |
| --- | --- | --- |
| Model 1 | Crude | No adjustment |
| Model 2 | Demographic-adjusted | Age, gender, race, education level, marital status, PIR |
| Model 3 | Fully adjusted | Demographic: Age, gender, race, education level, marital status, poverty-income ratio (PIR)  Anthropometric & Behavioral: Body mass index (BMI), smoking status  Comorbidities: Hypertension, diabetes  Laboratory Parameters: Serum triglycerides (TG), total cholesterol (TC), high-density lipoprotein cholesterol (HDL-C), low-density lipoprotein cholesterol (LDL-C), serum uric acid (SUA), serum creatinine (SCR), blood urea nitrogen (BUN), urinary albumin (UA), urinary creatinine (UCr) Nutritional Intakes: Vitamin B6, vitamin B12, calcium, magnesium, total energy, total fat |

Supplementary Table S4. Description of covariates included in each multivariable logistic regression model.
