# Supplementary material for: Riboflavin intake and kidney health: population evidence and mechanistic insights from NHANES and molecular docking analyses
Source: Ren Fail. 2026 Jan 25;48(1):2611520. doi: 10.1080/0886022X.2025.2611520 (PMC12836406; doi:10.1080/0886022X.2025.2611520)
Supplement: Supplementary Table S1.docx [file IRNF_A_2611520_SM9509.docx]

| Targets | RCSB IDs | (X, Y, Z) | size |
| --- | --- | --- | --- |
| CASP3 | 1NME | (36.2,93.5,18.3) | 14.7,15.1,14.9 |
| ERBB2 | 7PCD | (8.4,-9.0,-13.5) | 24.0,26.1,19.7 |
| MMP9 | 4XCT | (18.4,-17.1,19.7) | 15.4,20.5,18.1 |
| ICAM1 | 5MZA | (3.6,-5.8,-1.3) | 45.7,39.2,41.6 |
| ACE | 2XY9 | (21.0,5.9,-13.5) | 38.7,57.8,42.9 |

Supplementary Table S1. The values of co-ordinates and grid size of molecular docking.
